# Adolescents with prenatal cocaine exposure show subtle alterations in striatal surface morphology and frontal cortical volumes

**DOI:** 10.1186/1866-1955-4-22

**Published:** 2012-08-07

**Authors:** Florence Roussotte, Lindsay Soderberg, Tamara Warner, Katherine Narr, Catherine Lebel, Marylou Behnke, Fonda Davis-Eyler, Elizabeth Sowell

**Affiliations:** 1 Department of Neurology, University of California, Los Angeles, CA, USA; 2 Developmental Cognitive Neuroimaging Laboratory (DCNL), Department of Pediatrics, University of Southern California, Los Angeles, CA, USA; 3 Department of Pediatrics, University of Florida, Gainesville, FL, USA; 4 Laboratory of NeuroImaging, Department of Neurology, University of California, Los Angeles, CA, USA

**Keywords:** Prenatal drug exposure, Cocaine, Striatum, Frontal lobes

## Abstract

**Background:**

Published structural neuroimaging studies of prenatal cocaine exposure (PCE) in humans have yielded somewhat inconsistent results, with several studies reporting no significant differences in brain structure between exposed subjects and controls. Here, we sought to clarify some of these discrepancies by applying methodologies that allow for the detection of subtle alterations in brain structure.

**Methods:**

We applied surface-based anatomical modeling methods to magnetic resonance imaging (MRI) data to examine regional changes in the shape and volume of the caudate and putamen in adolescents with prenatal cocaine exposure (n = 40, including 28 exposed participants and 12 unexposed controls, age range 14 to 16 years). We also sought to determine whether changes in regional brain volumes in frontal and subcortical regions occurred in adolescents with PCE compared to control participants.

**Results:**

The overall volumes of the caudate and putamen did not significantly differ between PCE participants and controls. However, we found significant (*P* <0.05, uncorrected) effects of levels of prenatal exposure to cocaine on regional patterns of striatal morphology. Higher levels of prenatal cocaine exposure were associated with expansion of certain striatal subregions and with contraction in others. Volumetric analyses revealed no significant changes in the volume of any subcortical region of interest, but there were subtle group differences in the volumes of some frontal cortical regions, in particular reduced volumes of caudal middle frontal cortices and left lateral orbitofrontal cortex in exposed participants compared to controls.

**Conclusions:**

Prenatal cocaine exposure may lead to subtle and regionally specific patterns of regional dysmorphology in the striatum and volumetric changes in the frontal lobes. The localized and bidirectional nature of effects may explain in part the contradictions in the existing literature.

## Background

Cocaine is a central nervous system stimulant that binds to and blocks the activity of monoamine transporters, resulting in increased synaptic and extracellular levels of dopamine, norepinephrine, and serotonin [1]. The animal literature suggests that prenatal cocaine exposure (PCE) affects brain development in various ways, in particular through diverse neurochemical and vasocontrictive mechanisms, as well as through epigenetic changes in placental DNA associated with the disruption of the hypothalamic-pituitary-adrenal (HPA) axis, resulting in lasting emotional and behavioral dysregulation [2].

The neurobehavioral effects of PCE have also been documented in humans. In particular, children exposed to cocaine *in utero* exhibit more negative behavioral functioning [3,4] than unexposed controls, and experience difficulties with emotion regulation [5,6]. They make more errors during attention and response inhibition tasks than non-exposed controls [7,8]. They also show deficits in procedural learning, visual motor, and motor skills [9,10]. Less is known about the neurobehavioral phenotype of adolescents with prenatal cocaine exposure, though one study suggests impairments in incidental memory [11].

The functional magnetic resonance imaging (fMRI) literature of PCE in humans, though small, suggests the existence of various types of functional brain abnormalities in youth with prenatal exposure to cocaine. For example, an earlier perfusion fMRI study reported changes in global cerebral blood flow (CBF) in the PCE group compared to controls [12]. While one blood oxygen level-dependent (BOLD) fMRI study found only trend-level differences in functional brain activation during a non-spatial working-memory task between PCE participants and controls [13], two other BOLD fMRI investigations reported significant differences between groups. Specifically, one study found group differences in task-related activation during a response inhibition task [14], and another reported differences in activation patterns associated with emotion-memory interactions [5]. Finally, three recent studies found group differences in functional and/or effective connectivity between participants with PCE and controls [15-17].

The neuroimaging literature addressing the structural effects of prenatal cocaine exposure on human brain development has yielded more inconsistent findings. A diffusion tensor imaging (DTI) study found higher average diffusion coefficients in the PCE group in left frontal callosal and right frontal projection fibers, suggesting suboptimal white matter development in these regions, regardless of prenatal exposure to other drugs of abuse [18]. Another investigation reported white matter reductions in the volume of the corpus callosum and gray matter reductions in occipital and parietal lobes, in PCE participants compared to controls [19]. The amount of cocaine ingested by the mother during pregnancy predicted the area of the corpus callosum and remained significant after controlling for prenatal exposure to other drugs [19]. Two additional studies reported changes in subcortical structures, specifically decreased caudate volumes [12,20], and increased gray matter volumes in the amygdala [12] in youth with prenatal exposure to cocaine, though these studies did not control for other gestational drug exposures.

In contrast, there are several reports of negative findings with regards to structural brain differences in PCE. One study combining structural magnetic resonance imaging (sMRI) and magnetic resonance spectroscopy (MRS) found no structural or volumetric abnormalities in any brain region in the PCE group [21]. The authors did observe an increase in frontal white matter creatine levels in the exposed group compared to controls, but did not control for prenatal exposure to tobacco or alcohol [21]. Another whole-brain volumetric study found no significant structural differences between PCE participants and controls after controlling for exposure to other drugs of abuse, suggesting that none of the initially observed brain volume reductions in the exposed group could be attributed to the specific teratogenic effects of cocaine [22]. In addition, a recent DTI study which included prenatal tobacco exposure as a nuisance covariate reported no significant differences between adolescents with PCE and control participants in any subregion of the corpus callosum [23].

To help clarify these discrepancies in the structural neuroimaging literature, here, we investigated the neurological consequences of prenatal exposure to cocaine using methodologies that allow for the detection of more subtle alterations in brain structure. We applied advanced surface-based anatomical modeling methods to MRI data, in order to examine regional changes in the shape and volume of the caudate and putamen. In addition, we examined regional volumetric differences between participants with prenatal cocaine exposure and unexposed controls in several subcortical and frontal cortical regions of interest implicated by some, though not all prior studies as indicated above.

Because of the mechanisms of action of cocaine, we focused our surface-based analyses on dopamine-rich striatal regions. Although cocaine is less neurotoxic than other stimulant drugs of abuse (such as methamphetamine) and may act as an intrauterine stressor rather than as a direct toxin [2], animal models have shown that prenatal exposure to cocaine leads to changes in dopamine receptor activity and subcellular distribution [24], and alterations in dendritic spine density in striatal medium spiny neurons [25]. Thus, in this study, we hypothesized that prenatal cocaine exposure would be associated with subtle structural differences in the morphology of the caudate and putamen. Specifically, we predicted a positive correlation between levels of prenatal cocaine exposure and extent of structural abnormalities in striatal surface morphology.

In adolescents with prenatal cocaine exposure, we also hypothesized relationships between performance on neuropsychological tests and regional changes in striatal surface morphology. In particular, we expected that deformations in the dorsal caudate (part of the executive loop [26]) would correlate with decreased scores on tests of executive functioning (Stroop test, Trail Making test part B), whereas deformations in the putamen (part of the fronto-striatal motor loop [26]) would correlate with lower scores on a visuomotor task (Trail Making test part A).

We also investigated possible regional volumetric differences between groups in subcortical and frontal cortical regions. We chose to restrict our analyses to these particular regions of interest because previous neuroimaging studies of structural and/or metabolic brain abnormalities in humans with prenatal cocaine exposure suggested evidence for differences in these areas. That is, most of the published structural human studies with significant results reported group differences in frontal [18,21] or subcortical [12,20] structures. Thus, in this study, we expected that prenatal cocaine exposure would be associated with subtle volumetric differences in frontal and subcortical areas.

## Methods

### Participants

Forty volunteers, age range 14 to 16 years, including 28 adolescents with prenatal cocaine exposure and 12 unexposed controls were studied with structural MRI collected at the University of Florida. Study approval was granted by the University of Florida Institutional Review Board, and a federal Certificate of Confidentiality protects the confidentiality of the data. All participants were the offspring of women prospectively enrolled during pregnancy in a longitudinal cohort study of the developmental effects of prenatal cocaine exposure [27]. A separate informed consent from the child’s primary caregiver and assent by the child were obtained before the current study. Detailed drug histories covering the period from 3 months prior to gestation through birth were obtained for all mothers. Prenatal cocaine exposure was measured as the ratio of weeks of maternal cocaine use during pregnancy over weeks of gestation. The extent of PCE in exposed participants ranged from 0.04 to 1, with a mean ratio of 0.402 and a standard deviation of 0.25 (Table 1). 

**Table 1:** Demographic information and neuropsychological data for subjects by group

	**Control group (CON,*n*** **= 12)**	**Prenatal cocaine exposure group (PCE,*n*** **= 28)**	**Group differences**
Age (in whole years)	14.7 ± 0.49	14.8 ± 0.72	*P* = 0.439
Gender	7 girls/	18 girls/	*P* = 0.039^a^
	5 boys	10 boys	
Cocaine exposure (weeks of maternal cocaine use divided by weeks of gestation)	None	0.402 ± 0.25	*P* <0.001^a^
Tobacco exposure (average number of cigarettes per day)	0.020 ± 0.071	8.155 ± 7.79	*P* <0.001^a^
Alcohol exposure (average ounces per day)	0.007 ± 0.015	0.199 ± 0.371	*P* = 0.011^a^
Marijuana exposure (average number of joints per day)	0.003 ± 0.010	0.125 ± 0.367	*P* = 0.091^b^
Postnatal cocaine exposure (hair sample positive for cocaine at age 10.5 years and/or 12.5 years)	6 yes / 6 no	10 yes / 18 no	*P* = 0.030^a^
Total brain volume (in mm^3^)	1,582,350	1,559,794	*P* = 0.846
	± 188,214	± 177,613	
Word-color interference Stroop test, raw score	44.417 ± 9.54	39.857 ± 9.15	*P* = 0.605
Trail Making test, part A completion time (in seconds)	11.750 ± 4.37	12.786 ± 4.78	*P* = 0.882
Trail Making test, part B completion time (in seconds)	23.000 ± 7.86	29.643 ± 11.00	*P* = 0.360

Mean values are given with standard deviation.

^a^Significant differences between groups (*P* <0.05).

^b^Trend-level differences between groups (0.05 < *P* < 0.10).

In addition to neuroimaging data, biologic assays were available for participants (hair samples, tested for cocaine at ages 10.5 years and 12.5 years), as well as neuropsychological data. Participants were administered a battery of tests at the time of scanning, which included a word-color interference Stroop test and a Trail Making test. We chose to examine these two particular measures of neurocognitive function in order to investigate relationships between the morphology of striatal structures in the executive and motor loops and tests of response inhibition (Stroop test), task switching (Trail Making test part B) and visuomotor function (Trail Making test part A).

### Image acquisition

Neuroimaging data were collected on a Philips 3 T Achieva MRI scanner. Conventional MRI sequences (axial T2 multishot turbo field echo) were obtained to detect possible confounding pathology. Volumetric T1-weighted image acquisition used a multishot gradient spin echo pulse sequence with 8.1-ms TR, 3.7-ms TE, 240 × 240 × 234 matrix, 1 mm isotropic voxel size, and an acquisition time of 10 min, 14 s.

### Image preprocessing and processing

#### Surface-based analyses

For the surface-based analyses, each brain volume was corrected for radiofrequency field inhomogeneities [28] and placed into the standard coordinate system of the ICBM-305 average brain volume using a three-translation and three-rotation rigid-body transformation [29]. This procedure corrects for differences in head alignment between subjects to ensure that region of interest measurements are not influenced by different brain orientations between subjects [29].

**Figure 1:**
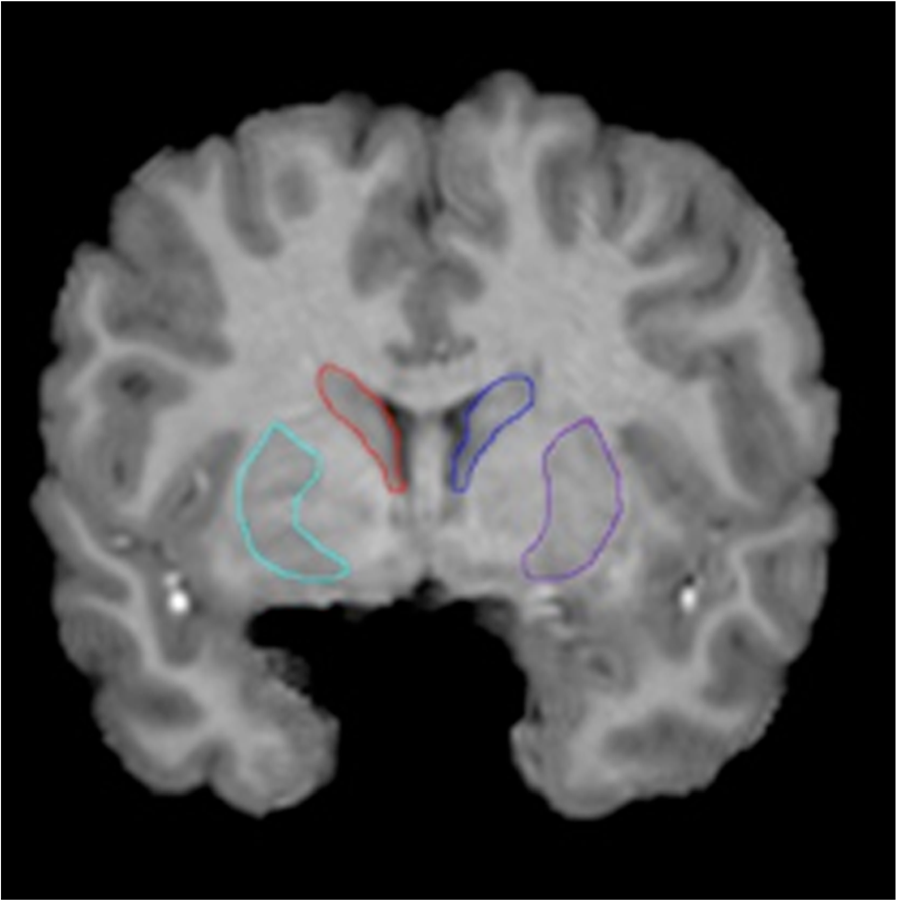
**Region of interest delineation.** The left and right caudate and putamen were manually delineated on contiguous coronal slices following a detailed protocol devised by the investigators.

The methods for surfaced-based image analysis have been described in detail elsewhere [30-32]. Briefly, two investigators blind to exposure status (FFR and LS) devised a detailed manual tracing protocol for the caudate and putamen, which included directions about the order and direction of tracing, numerous visual aids to facilitate the identification of anatomical landmarks, and precise instructions for dealing with scan artifacts. Striatal contours were then manually outlined by the same two investigators on contiguous coronal slices for every subject (Figure 1). High intra-rater and inter-rater reliabilities (intraclass correlations >0.95) were established based on independent blinded measurement of six scans used in this study. Subsequently, manually derived contours were transformed into 3D parametric surface mesh models with normalized spatial frequency of the surface points within and across brain slices. Each structure was made into a parametric grid containing 100 × 150 grid points or surface nodes. This step ensures precise comparison of anatomy between subjects at each surface point of the structure. A 3D medial curve was computed along the long axis for the surface model of each structure and radial distance measures (distance from the medial core to the surface) were estimated and recorded at each corresponding surface point. These values were used to generate individual distance maps, which were combined to produce correlation maps allowing for visualization of the relationships between striatal morphology and (1) levels of prenatal cocaine exposure, and (2) neuropsychological test scores in exposed participants. In all analyses, quantitative measures of prenatal exposure to tobacco, alcohol, and marijuana were included as nuisance covariates, as well as sex, cube root of total brain volume, and cocaine use by adolescent participants themselves. Since this method estimates radial distance measures, and distance from the medial core to the surface is a 1D measure, cube root of brain volume was used in place of total brain volume (a 3D measure), in order to make the units comparable. 

#### Volumetric analyses

Preprocessing and definition of cortical and subcortical gray matter regions on structural images were conducted in the UCLA Laboratory of Neuro Imaging (LONI) Pipeline Processing Environment [33-35] and using FreeSurfer’s automated brain segmentation software (FreeSurfer 4.0.5, http://surfer.nmr.mgh.harvard.edu), as described in the work of Fischl and Dale [36-38]. We obtained volume measurements of seven subcortical brain regions (thalamus, caudate, putamen, pallidum, hippocampus, amygdala, and ventral diencephalon) as well as six frontal cortical regions (caudal middle frontal cortex, rostral middle frontal cortex, lateral orbitofrontal cortex, medial orbitofrontal cortex, superior frontal cortex, and frontal poles). During preprocessing, high-resolution T1-weighted image acquisitions for each participant were visually inspected for motion artifacts by a trained rater based on a 5-point Likert scale illustrating the severity of motion effects. No participants were rejected due to motion artifacts; however, one subject was rejected due to poor gray -white matter contrast, and another subject was rejected because the raw image data files appeared corrupted. In all remaining participants (*n* = 40), the T1-weighted images were brain extracted, and gray-white matter boundaries were automatically delineated. All brain extractions were inspected visually and corrected manually as needed.

Volumes for the seven subcortical and six frontal cortical regions of interest, as well as total intracranial volumes were calculated using FreeSurfer’s automatic quantification of cortical and subcortical structures. Procedures are described in detail elsewhere [38]. In summary, a neuroanatomical label was assigned to each voxel in an individual’s structural MRI based on probabilistic information estimated from a manually labeled training set. This manually labeled training set is a result of validated methods from the Center of Morphometric Analysis ( http://www.cma.mgh.harvard.edu). To disambiguate the overlap in intensities between different anatomical structures, FreeSurfer utilized spatial information. Two transformations were performed. First, an optimal linear transformation was carried out by maximizing the likelihood of the native image given a manually labeled atlas. Second, a non-linear transformation was executed on the output of the prior registration step. Finally, a Bayesian parcellation was conducted by using prior spatial information [39,40]. At the end of this processing stream, three probabilities were calculated for each voxel: (1) the probability of the voxel belonging to each of the label classes, based on its location, (2) the neighborhood function, used to determine the likelihood that the voxels belong to a class, based on the classification of neighboring voxels, and (3) the result of the probability distribution function for each voxel based on its intensity.

The accuracy of this technique was shown to be similar to manual methods. The automated segmentations have been found to be statistically indistinguishable from manual labeling [38]. Being completely automated, Freesurfer volume estimates are highly reliable. Nonetheless, in the current study, each brain image was visually inspected for validity of all regions by a single trained blind rater. In over one-third of subjects, the segmentations of the caudate and putamen were judged unsatisfactory, due to pulsation artifacts around the striatum on the high-resolution T1-weighted images. Therefore, the volumes of the caudate and putamen were calculated from the manually derived contours (obtained in the surface-based analyses) for all subjects (*n* = 40), and these values were used in place of the FreeSurfer outputs in all statistical analyses.

### Statistical analyses of demographic, neuropsychological, and volumetric data

Statistical analyses were conducted using SYSTAT 12.0 and SPSS 20.0. Group differences in age, total brain volume, neuropsychological test performance, and prenatal exposure to tobacco, alcohol, and marijuana were evaluated using two-sample independent t-tests. Group differences in gender and postnatal cocaine exposure were assessed with a Pearson Chi-Square Test.

In volumetric analyses, group differences in regional brain volumes were evaluated using separate one-way ANOVA tests for each individual region of interest. In all analyses, prenatal exposure to cocaine was modeled as the independent variable, while the volume (in mm^3^) of the region of interest was used as the dependent variable. All analyses included the following covariates: prenatal exposure to alcohol, tobacco, and marijuana, in addition to sex, total brain volume, and cocaine use by participants themselves (as measured by a positive hair sample at 10.5 and/or 12.5 years of age). Two-way ANOVAS were subsequently performed in order to examine possible interactions between pre- and postnatal cocaine exposure.

Associations between neuropsychological test scores and levels of prenatal cocaine exposure were investigated with multiple regression analyses using the following equation: Performance = Constant + Level of Prenatal Cocaine Exposure + Prenatal Alcohol Exposure + Prenatal Tobacco Exposure + Prenatal Marijuana Exposure + Sex + Cocaine Use by Participants (as measured by a positive hair sample at 10.5 and/or 12.5 years of age).

## Results

### Demographics

Demographic descriptors are reported in Table 1. The PCE and CON groups did not differ from each other in age (*P* = 0.439) but showed a significant difference in gender distribution (*P* = 0.039), with the exposed group comprising more girls. The two groups significantly differed from each other in prenatal exposure to tobacco (*P* <0.001) and alcohol (*P* = 0.011), with the PCE group showing significantly higher levels of exposure to these drugs than participants in the CON group. We also reported a trend-level difference in prenatal exposure to marijuana (*P* = 0.091) and a significant difference in postnatal exposure to cocaine (*P* = 0.030) assessed by cocaine-positive hair samples at age 10.5 years and/or 12.5 years. The PCE group tended to have higher levels of prenatal marijuana exposure, and while the CON group comprised an equal number of participants with and without postnatal cocaine exposure, the PCE group included almost twice as many non-users as users. The two groups did not differ in total brain volume (*P* = 0.846). Nevertheless, in all analyses presented below, total brain volume was used as a covariate along with sex, postnatal cocaine exposure, and prenatal exposure to other drugs of abuse in order to eliminate any variance due to possible disparities in overall brain volume within groups.

### Surface-deformation analyses

#### Main effects of prenatal cocaine exposure

The overall volumes of the caudate and putamen did not significantly differ between participants with prenatal cocaine exposure (the PCE group) and controls (the CON group). In addition, differences in caudate and putamen morphology between these two groups were non-significant after regressing out prenatal exposure to other drugs of abuse, total brain volume, sex, and cocaine use, even when prenatal tobacco exposure was not included as a nuisance covariate. However, 3D surface statistics revealed significant (*P* <0.05, uncorrected) effects of the amount of prenatal exposure to cocaine on regional patterns of striatal morphology across all participants.

Higher levels of prenatal cocaine exposure were associated with contraction of striatal surfaces in the ventromedial and in the dorsal caudate, and some regions of expansion in the ventrolateral caudate. These effects were stronger in the left caudate (Figures 2.2 and 2.3). Similar but less pronounced results were observed in the right caudate (data not shown).

**Figure 2:**
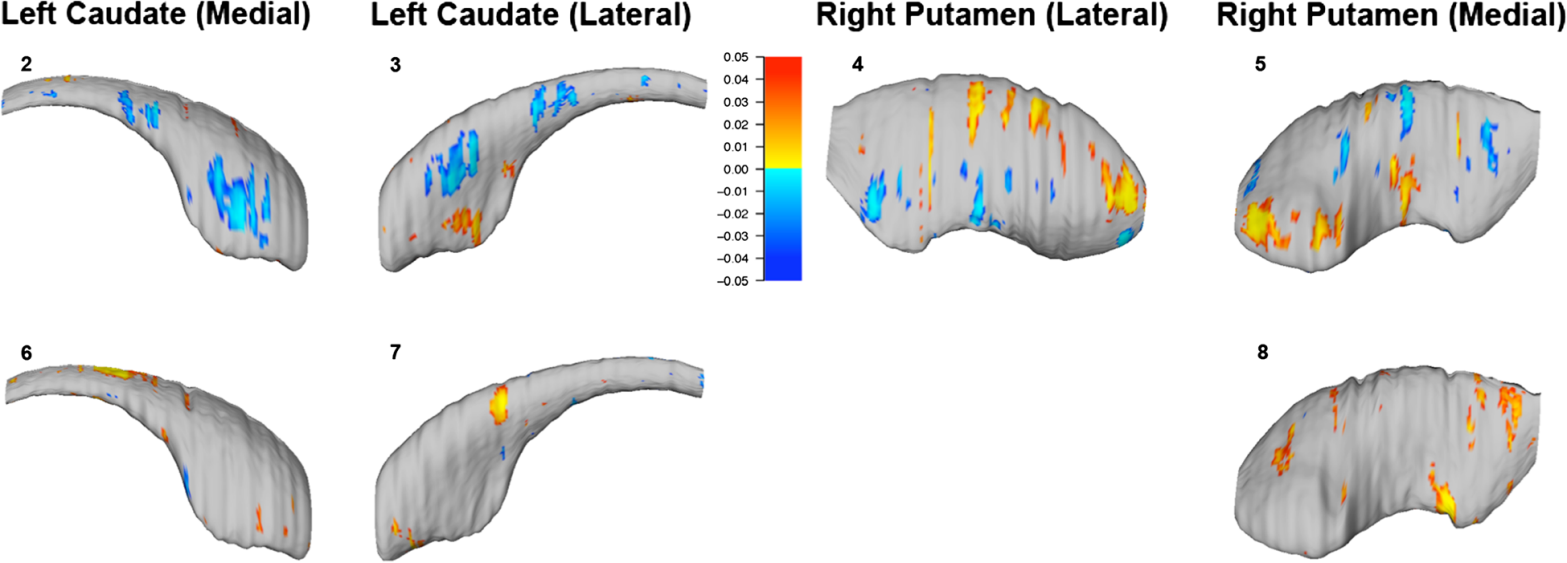
**2, 3, 4, and 5: Uncorrected surface maps depicting relationships between levels of prenatal cocaine exposure and regional deformations of striatal surface (*n*** **= 40).** Blue-to-light-blue shading indicates regions where higher levels of prenatal cocaine exposure are associated with contraction of striatal surfaces. Red-to-yellow shading displays regions where higher exposure levels are associated with expansion of the surfaces. For all statistical maps, the color bar encodes the uncorrected *P* values (*P* <0.05) for the observed effects. **6, 7, 8:Uncorrected surface maps depicting relationships between neuropsychological scores (6: Stroop, 7: Trails A, 8: Trails B) and regional deformations of striatal surface in exposed subjects (*n* = 28)**. Red-to-yellow shading displays regions where higher scores (better performance on the Stroop test but longer response times on the Trails test) are correlated with larger regional striatal volumes. Few negative correlations were observed in these analyses. For all statistical maps, the color bar encodes the uncorrected *P* values (*P* <0.05) for the observed effects.

Greater prenatal cocaine exposure was associated with contraction of striatal surfaces in the posterior putamen and in the ventrolateral and dorsomedial putamen. Higher levels of prenatal cocaine exposure were associated with large areas of expansion in the anterior putamen, as well as in the dorsolateral and ventromedial putamen. These effects were stronger in the right putamen (Figures 2.4 and 2.5). Similar but less pronounced results were observed in the left putamen (data not shown). Comparable correlational maps in terms of strength and extent were obtained in the left and right caudate and putamen when prenatal tobacco exposure was not included as a nuisance covariate (data not shown). These results did not remain significant after correcting for multiple spatially correlated comparisons using permutation testing.

#### Neuropsychological correlates of striatal morphology

Following these initial analyses, we examined the two ROIs that showed the most significant effects (left caudate and right putamen), and investigated relationships between neuropsychological test scores and regional deformations of the striatal surface in exposed subjects. Specifically, we first examined correlations between measures of executive functioning and regional deformation of the left caudate surface. Higher scores (better performance) on the Stroop test (Figure 2.6) were associated with larger volume in subregions of the dorsal caudate, while longer response times (lower performance) on part B of the Trail Making test (Figure 2.7) correlated with larger volume in other parts of the dorsal caudate.

We also investigated correlations between a measure of visuomotor functioning and regional deformation of the right putamen surface. Longer response times on part A of the Trail Making test were associated with larger volume of the medial putamen, mostly in the posterior region (Figure 2.8). None of these results remained significant after performing permutation testing in order to correct for multiple spatially correlated comparisons.

### Volumetric analyses

In these analyses, we examined group differences in regional brain volumes between the PCE group and the CON group. As in the surface-deformation analyses, in addition to sex, total brain volume, and cocaine use by the participants themselves, we also used prenatal exposure to alcohol, tobacco, and marijuana as nuisance covariates in all analyses in an attempt to detect the specific effects of prenatal cocaine exposure.

Consistent with the surface-based analyses in which we found no significant group differences in overall volumes of the caudate and putamen based on manually-derived contour, here we observed no significant changes in the volume of any subcortical region of interest in the PCE group compared to the CON group in analyses based on automated segmentation. However, we detected group differences in the volumes of some frontal cortical regions.

**Figure 3:**
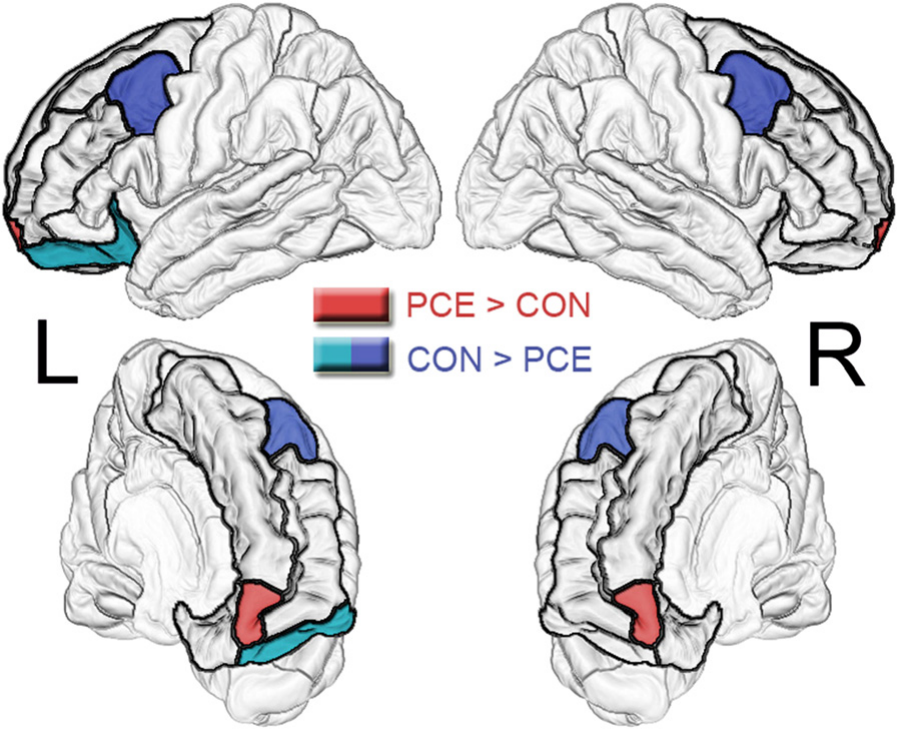
**Group differences in frontal cortical brain volumes (uncorrected results).** Blue and green shading indicates regions where the PCE group showed decreased volumes compared to controls (*P* <0.05) after controlling for prenatal exposure to tobacco, alcohol, and marijuana as well as sex, total brain volume, and drug use by participants. Red shading indicates regions where the PCE group showed trends for increased volumes compared to controls (*P* <0.10), using the same covariates. Thicker black contours delineate all of the frontal regions of interest that were examined in these analyses.

We observed significant (uncorrected) reductions in regional volumes in the left (*P* = 0.046) and right (*P* = 0.036) caudal middle frontal cortices, and in the left lateral orbitofrontal cortex (*P* = 0.048), in participants with prenatal cocaine exposure compared to controls (Figure 3). On the other hand, in the left (*P* = 0.072) and right (*P* = 0.098) frontal poles, we observed trend-level increases in regional brain volumes in the PCE group compared to controls (Figure 3).

We found no significant main effects of postnatal cocaine exposure (cocaine use by participants) on the volume of any frontal cortical region, and there were no significant interactions between pre- and postnatal cocaine exposure. Moreover, neuropsychological performance on the word-color interference Stroop Test, and on parts A and B of the Trail Making Test did not differ significantly between the PCE and control groups (Table 1); and there were no significant correlations between levels of prenatal cocaine exposure and neuropsychological test scores, after co-varying for prenatal exposure to other drugs of abuse, sex, and postnatal cocaine exposure.

## Discussion and conclusions

Taken together, these results suggest that prenatal cocaine exposure may lead to regionally specific patterns of morphological changes in the striatum and subtle volumetric differences in certain frontal cortical regions. The most significant finding in our analyses of caudate morphology was an association between levels of prenatal cocaine exposure and surface contraction in the ventromedial and dorsolateral caudate. The ventromedial caudate is part of the lateral orbitofrontal striatal loop, which is involved in the regulation of emotion and social behavior [26]. Interestingly, we also reported regional volume changes in the left lateral orbitofrontal cortex in the PCE group compared to controls. The dorsolateral caudate, on the other hand, is part of the executive loop associated with higher-order cognitive functions [26], and we also found local volumetric differences in the frontal poles, which play a role in spatial working memory, response inhibition [41], and the evaluation of self-generated [42] and goal-directed [43] decisions. Therefore, the findings presented here may represent some of the neural correlates of the difficulties in emotional regulation [5,6] and impairments in attention, response inhibition [7], and visuospatial working memory [44] that have been reported in children with prenatal cocaine exposure.

The putamen is part of the fronto-striatal loop involved in motor control [26]. Most premotor area projections are directed to the medial putamen, and most supplementary motor area projections terminate in the posterior putamen [45], and in both subregions we found that greater prenatal cocaine exposure was associated with a contraction of striatal surfaces. In addition, we observed significant reductions in regional volumes in the bilateral caudal middle frontal cortices in the PCE group compared to controls, and the caudal part of the middle frontal gyrus corresponds to premotor brain areas. Therefore, it is possible that these findings may be related to the deficits in fine motor coordination that have been reported in this population [10].

While the direction of changes in striatal surface structure were not predicted *a priori*, it should be noted that prenatal cocaine exposure was associated with surface contraction in some subregions, and expansion in others. Though the biological mechanisms contributing to these findings remain unclear, the bidirectional nature of regional effects may explain why overall differences in striatal volume were not detected in this study between exposed and control participants in either the surface-based or the volumetric analyses. Similarly, while we did not have specific predictions about the direction of changes in regional frontal cortical volumes, prenatal cocaine exposure was shown to be associated with volume reductions in some frontal subregions, and increased volumes in others. The reasons for this discrepancy remain unclear, but the localized and bidirectional nature of frontal cortical effects may explain in part why certain neuroimaging studies found significant structural differences in the frontal lobes, while others reported negative results.

Consistent with our predictions, we observed very narrowly localized correlations between measures of executive functioning and regional deformation of the dorsal caudate surface. However, the specific areas where this association was significant did not correspond exactly to the subregions of the dorsal caudate where higher levels of prenatal exposure were correlated with greater dysmorphology. The specific areas of the dorsal caudate showing correlations with measures of executive functioning also differed by task: they were more superior and medial for the Stroop test than for part A of the Trail Making test.

As predicted, we also observed a marginally significant correlation between a measure of visuomotor performance and regional deformation of the putamen surface, which was significant in the medial and posterior putamen, where most premotor and supplementary motor area projections terminate [45], suggesting a possible association between neurological and behavioral abnormalities.

However, while these results suggest that prenatal exposure to cocaine may affect the morphology of the striatum and regional frontal lobe volumes, it is important to keep in mind that the effect sizes were small for all of the results reported here, and that the surface-deformation and volumetric maps were not corrected for multiple spatially correlated comparisons. Nevertheless, the fact that we reported subtle changes consistent with findings from the animal literature and with our *a-priori* hypotheses, suggests that these differences may be due in part to the specific effects of prenatal cocaine exposure.

We found no association between PCE and neurological test scores, which suggests that brain structure may be a more sensitive biomarker to levels of prenatal cocaine exposure than neuropsychological test performance. This is consistent with findings from the animal literature, suggesting that cognitive tests may be more sensitive to the pattern of maternal consumption than to the amount of cocaine intake, even in the presence of neurobiological alterations [46]. Despite showing evidence for abnormal neuronal migration and cortical lamination as well as neurochemical differences, rhesus monkeys with both ‘high doses’ and ‘low doses’ of PCE do not significantly differ from controls in cognitive performance, whereas monkeys in the ‘escalating dose’ group show impairments [46].

It should be noted that we did not have data about participants’ use of other drugs, thus we cannot exclude the possibility that postnatal exposure to other substances of abuse may have affected the findings. Another important limitation is that neuroimaging data was available for only 12 adolescent controls. Though we cannot rule out the eventuality that a larger control group would have allowed for the detection of slightly more robust group differences, in the context of the existing literature, these findings support the notion that the effects of PCE on brain structure may be quite subtle. An alternative explanation to findings of modest effects in the current study may be that the marginally significant alterations observed simply reflect relatively minor consequences of PCE on brain development. Some publications suggested that cocaine may be a relatively weak teratogen with few observable neurological or behavioral consequences in humans [47], unlike other common substances of abuse during pregnancy, which have been more convincingly linked to psychopathology risk, such as alcohol [48] and nicotine [49].

The small effect sizes observed here as well as regional differences in the direction of effects may help explain why prior investigations of the consequences of prenatal cocaine exposure on brain structure have yielded somewhat conflicting results. Although animal studies of PCE, particularly studies of non-human primates, clearly demonstrate the potential of prenatal cocaine exposure to interfere with brain development at a cellular and biochemical level in various brain regions [24,25,47,50-52], they also suggest that the types and severity of PCE effects largely depend on the route, dose, gestational period, and pattern of consumption [46]. These could be additional contributing factors to the discrepancies in the existing human neuroimaging literature, and to the small effect sizes reported here.

Thus, it is important that future neuroimaging studies of prenatal cocaine exposure with larger samples collect information about adolescent participants’ use of other substances, specific patterns and timing of maternal cocaine consumption, and aim to integrate observations from different brain imaging modalities. This will help determine how structural, metabolic, and functional brain abnormalities resulting from PCE relate to real-life difficulties these children face outside the scanner, as subtle neurological changes may very well be associated with important behavioral, cognitive, or emotional impairments.

Although several promising psychosocial prevention strategies for pregnant women addicted to cocaine have been identified [53], effective remediation strategies and treatments for prenatally exposed children remain to be developed. The improvement of such strategies will require that we gain a better understanding of the specific, localized and perhaps subtle neurological abnormalities resulting from prenatal cocaine exposure in order to facilitate the translation of research findings to clinical practice.

## Competing interests

The authors declare that they have no competing interests.

## Authors’ contributions

FR analyzed and interpreted the data and was the main writer of the manuscript. LS helped with data processing and analysis. TW helped with data collection and data interpretation. KN helped with technical aspects of data analysis and data interpretation. CL helped with data interpretation and helped revise the manuscript. MB helped with data collection and interpretation. FDE helped with data collection and interpretation. ERS was the principal investigator, who supervised the whole project, and helped write the manuscript. All authors read and approved the final manuscript.
